# 

**Published:** 1863-01

**Authors:** 


### INDEX

TO

### HALL'S JOURNAL OF HEALTH.

### VOL. X. 1863.

PAGE

Apprentices, .................... 31

Anecdotes, .................... 111, 112

Agreeableness, .................... 262

Autocrat's Death, .................... 269

Baldness, .................... 22

Bible Confirmations, .................... 50, 79

Burns, .................... 85

Bilious Diarrhea, .................... 163

Blind Boy, .................... 237

Catching Cold, .................... 13
Corns, .................... 19
Cherished Flower, .................... 51
'Cute Things, .................... 66
Clay, Henry, .................... 83
Cures, .................... 84
Coffee, .................... 91, 122
Changing Clothing, .................... 86, 115
Constipation, .................... 138
Cholera, .................... 161
Central Park, .................... 167
Croup, .................... 177
Children, .................... 205
Children's Feet, .................... 282
Clerical Support, .................... 229
Curious Epitaph, .................... 231
Cottage, Sea, .................... 238
Consumption Detected, .................... 245
Cancer, .................... 267

Dress, .................... 17
Daughters, .................... 30
Dirt, .................... 47
Dying, .................... 58, 126
Drowning, .................... 156
Dieting, .................... 156
Diphtheria, .................... 159
Dysentery, .................... 162
Disinfectants, .................... 164

PAGE

Eating, .................... 9, 116, 216
Eyesight, .................... 101
Emanations, .................... 155
Exercise, .................... 219
Evermore, .................... 243
Eloquence, .................... 265

Farmer Health, .................... 5
Farmers' Wives Overtaxed, .................... 35
Facts, Interesting, .................... 72

Great Eaters, .................... 216
Gruels and Soups, .................... 283

Housewifery, .................... 20
Household Vermin, .................... 92
Haunted Houses, .................... 95
Household Economy, .................... 142
Head Vermin, .................... 182

Insanity, .................... 218
Ill-Nature, .................... 257

Life Wasted, .................... 89
Loose Bowels, .................... 160
Logic Run Mad, .................... 217
Lung-Measurement, .................... 221
Loss of Children, .................... 205, 265

Mothers' Influence, .................... 28
Making Money, .................... 176
Medical Items, .................... 214, 284
Month Malign, .................... 215
Manners, .................... 261
Music Lessons, .................... 263

Natives and Foreigners, .................... 60
Nicholas of Russia, .................... 269
Notices, .................... 285

PAGE
One Acre,........................113

Paine, Thomas,................... 52
Poverty and Disease,.............. 93
Parental Corrections,.....191, 232, 253
Potatoes,........................ 21
Premature Deaths,................ 25
Pulpit Power,.................... 48
Public Schools,.................. 61
Poisons,......................... 90
Philosophy,......................111
Piles,...........................119
Purgative Medicines,.............127
Physicians,......................143
Pew System,......................149
Physiological Items,.............165
Physiology of Worship,.......213, 266
Poetry, its Hygiene,...............

Responsibility of Writers,......... 27

Recreation,...................... 71

Reformers,....................... 74

Randolph, John,.................. 76

Rosetta Stone,................... 79

Religious Papers, Age of,......... 81

Resignation,.....................125

Raising Children,................205

Revenges,........................256

Shoes for Winter,................ 19
Success in Life,................. 25
Sabbath Observance,..........57, 166
Spot, The One,................... 67
Schools,.....................61, 88
Sad Reflection,.................. 82
Soldiers,........................ 93

PAGE
Surgery,.........................102
Specifics,.......................112
Spring-Time,.....................114
Sibley, Major,...................130
Summer Drinks,...................158
Summer Recreations,..............204
September,.......................215
Summer Mortality,................220
Spirometry,......................221
Sill, Door,......................239
Style,...........................263
Stammering,......................281
Soups,...........................283

Temper Controlled,............... 29
Thorburn, Grant,................. 55
Tumors,..........................105
Temper and Health,...............264
The Young Suicide,...............276

Unskilled Labor.................. 31

Ventilation,..................... 65

Vital Capacity,..................245

Wise Workers,.................... 57
Woolen Clothing,................. 86
White Washes,.................... 87
Worth Remembering,...............212
Wife Dying,......................241
Wedding-Ring,....................242
Wealth,..........................259
Weather,.........................268

Youth and Age,...................240

